# Phenotypic covariance structure and its divergence for acoustic mate attraction signals among four cricket species

**DOI:** 10.1002/ece3.76

**Published:** 2012-01

**Authors:** Susan M Bertram, Lauren P Fitzsimmons, Emily M McAuley, Howard D Rundle, Root Gorelick

**Affiliations:** 1 Department of Biology, Carleton UniversityOttawa, Ontario, K1S5B6, Canada; 2 Current Location: Department of Biological Sciences, Simon Fraser UniversityBurnaby, V5A1S6, British Columbia; 3 Department of Biology, University of OttawaOttawa, Ontario, K1N6N5, Canada

**Keywords:** Acoustic signaling traits, crickets, *Gryllus*, mate attraction, matrix dimensionality, multivariate, phenotypic variance–covariance matrix, P matrix

## Abstract

The phenotypic variance–covariance matrix (P) describes the multivariate distribution of a population in phenotypic space, providing direct insight into the appropriateness of measured traits within the context of multicollinearity (i.e., do they describe any significant variance that is independent of other traits), and whether trait covariances restrict the combinations of phenotypes available to selection. Given the importance of P, it is therefore surprising that phenotypic covariances are seldom jointly analyzed and that the dimensionality of P has rarely been investigated in a rigorous statistical framework. Here, we used a repeated measures approach to quantify P separately for populations of four cricket species using seven acoustic signaling traits thought to enhance mate attraction. P was of full or almost full dimensionality in all four species, indicating that all traits conveyed some information that was independent of the other traits, and that phenotypic trait covariances do not constrain the combinations of signaling traits available to selection. P also differed significantly among species, although the dominant axis of phenotypic variation (*p*_max_) was largely shared among three of the species (*Acheta domesticus*, *Gryllus assimilis*, *G. texensis*), but different in the fourth (*G. veletis*). In *G. veletis* and *A. domesticus*, but not *G. assimilis* and *G. texensis*, *p*_max_ was correlated with body size, while *p*_max_ was not correlated with residual mass (a condition measure) in any of the species. This study reveals the importance of jointly analyzing phenotypic traits.

## Introduction

Males often use multicomponent sexual signals to attract mates (Møller and Petrie 2002; Candolin 2005; Hebets and Papaj 2005). These signals can be bright, aromatic, or noisy (Andersson 1994). Females, in turn, often exhibit distinct preferences for specific components of male sexual signals (Andersson 1994; Candolin 2005; Hebets and Papaj 2005). Female preferences for both intermediate and extreme phenotypes are common in many anuran and insect mating systems (Gerhardt 1994b). Preferences for intermediate phenotypes result in stabilizing selection, whereas preferences for extreme male phenotypes result in directional selection (Paterson 1982, 1985; Butlin et al. 1985; Walker and Forrest 1989; Gerhardt 1991; Ryan and Keddy–Hector 1992; Gerhardt 1994a; Gerhardt and Huber 2002). Because multicomponent sexual signals can experience different selection regimes, female preferences can result in mating signals experiencing relatively complex selective landscapes. Understanding how signaling components covary can provide insight into whether certain trait combinations are available to selection.

There is a long history to the idea that relationships among traits can impose evolutionary constraints (Darwin 1871; Fisher 1930; Dickerson 1955; Lande 1979; McGuigan and Blows 2007). The phenotypic variance–covariance matrix (P) describes the variance–covariance structure of a suite of phenotypic characters. As such, P helps define the multivariate phenotype available to selection (Lande and Arnold 1983; Steppan 1997; Arnold and Phillips 1999; McGuigan and Blows 2007). The phenotypic variance–covariance structure is of interest, because it (1) describes the multivariate distribution of the population in phenotypic space, (2) informs us if the matrix is less than full dimensionality (has fewer linearly independent dimensions than measured traits), indicating combinations of traits that do not exist in the population and are thus unavailable to selection, (3) may place an upper bound on the dimensionality of the underlying genetic variance–covariance matrix (G) that shapes and potentially constrains the response to selection, (4) may provide additional information about the structure of G, and (5) provides insight into whether the traits are appropriately chosen, indicating whether they represent axes of phenotypic variance that are at least partly independent of other traits. P matrices can thus provide valuable information about the phenotypic space of multicomponent signals, provided they are built using biologically relevant traits. Traits should be selected to avoid multicollinearity (high correlations among traits; Lande and Arnold 1983; McGuigan and Blows 2007). Traits should also describe variance that is independent from the variance described by other traits (Lewontin 1978; Fristrup 2001; McGuigan and Blows 2007), because there is no evolutionary information gleaned from traits that are perfectly correlated with one another. Recent statistical advances have enabled researchers to explicitly test for matrix dimensionality. Statistically testing for dimensionality of P can provide insights into whether traits used to characterize the phenotype fit the above criteria (McGuigan and Blows 2007).

To date, only one study has explicitly tested the dimensionality of P. McGuigan and Blows (2007) chose 10 traits representative of aspects of wing size and shape in *Drosophila bunnanda*. Using a repeated measures approach, they estimated dimensionality of P and thereby inferred potential constraints on wing size and shape (McGuigan and Blows 2007). Their P matrices were of full dimensionality, suggesting that selection could, in theory, act upon any combination of these trait, driving the population toward an adaptive peak regardless of the position of the optimum. McGuigan and Blows (2007) intentionally chose traits that would maximize the probability of observing a P matrix of full dimensionality, and it remains to be seen whether similar results would be obtained for behavioral instead of morphometric traits. Comparisons of P across species may also provide understanding about how species differ in how they experience selection and permit tests of the processes underlying phenotypic divergence.

We tested the dimensionality of P for a set of behavioral traits involved in cricket long-distance acoustic mate attraction. Cricket mating signals have surfaced as an important model system in evolutionary biology and behavioral ecology (Cade 1975; Harrison 1980; Hedrick 1986, 1988; Bertram 2002b; Hunt et al. 2004; Simmons 2005; Bertram et al. 2009; Bertram et al. 2010; Drayton et al. 2010; Logue et al. 2010; Rodríguez–Muñoz et al. 2010). We performed repeated measures of seven mating signals for replicate individuals across four species. Six of these traits appear to be indicative of signaling quality, while the seventh is indicative of signaling quantity.

Most research on cricket mate signaling has either explored factors influencing signaling quality (Zuk et al. 1998; Kolluru 1999; Wagner and Hoback 1999; Hedrick 2000; Holzer et al. 2003; Simmons 2004; Judge et al. 2008; Whattam and Bertram 2011) or factors influencing signaling quantity (Bertram 2000, 2002a; Bertram and Bellani 2002; Hunt et al. 2004; Orozco and Bertram 2004; Bertram 2007; Judge et al. 2008; Bertram et al. 2009). It is largely unknown whether signaling quality is correlated with signaling quantity (but see Bertram et al. 2011b; Whattam and Bertram 2011). Given that conspicuous signaling can impose dramatic costs both in terms of energetic demands and predation risks (Prestwich and Walker 1981; Prestwich 1994; Hoback and Wagner 1997; Basolo and Alcaraz 2003), males that can maximize the conspicuousness of their mating signals in the face of these costs are thought to exhibit higher genetic quality (Andersson 1994). Under this honest signaling scenario, males in good condition might simultaneously maximize several components of their signals’ conspicuousness (Rowe and Houle 1996; Cotton et al. 2006). We therefore used a multivariate approach to determine whether signaling traits reflecting quality are correlated with each other and with signaling traits reflecting quantity. We also determined whether cricket mating signals reflect differences in body size and/or condition because the honest signaling scenario suggests that sexual signals exhibit condition-dependent expression (genetic capture hypothesis; Rowe and Houle 1996; Kotiaho et al. 1997; Tomkins et al. 2004)

To explore the covariances between signaling traits, how they might be influenced by condition, and how they might be evolutionarily constrained, we quantified the phenotypic variance–covariance matrix for each of four cricket species. Using these P matrices, we were then able to test the following three hypotheses:

1. Different species have different P matrices.
2. P matrices have full dimensionality.
3. The dominant axis of variation in P (*p*_max_) is correlated with body size and/or condition in each species.

We studied European house crickets (*Acheta domesticus*), Jamaican field crickets (*Gryllus assimilis*), Texas field crickets (*G. texensis*), and spring field crickets (*G. veletis*) because these four species are likely to be influenced by different selective regimes. *Gryllus texensis* are often stalked by acoustically orienting parasitoid flies (Tachinidae; *Ormia ochracea*) and may experience intense selection to not signal (Cade 1975; Wineriter and Walker 1990; Robert et al. 1992; Adamo et al. 1995). By contrast, *A. domesticus* have been reared in captivity for several decades and therefore may have experienced very different selection on long-distance signaling compared to the three *Gryllus* species. *Gryllus assimilis* and *G. veletis* likely lie somewhere in the middle of this continuum, as they are not stalked by *O. ochracea*, yet they are bound to experience very different sexual selection than laboratory-reared *A. domesticus*.

## Material and Methods

### Collections and colonies

We collected several hundred adult *G. texensis*, *G. assimilis*, and *G. veletis* from fields in their natural habitats in 2008. *Gryllus veletis* were collected in May and June in the Ottawa, Ontario, Canada area. *Gryllus texensis* and *G. assimilis* were collected in September and October in the area from Austin to Smithville, Texas. All three of these species of field cricket were visually and acoustically located and then hand captured between the hours of 2100 and 0200. We obtained *A. domesticus* from a local breeder in Ottawa, Ontario, Canada.

Cricket colonies were housed in a greenhouse at Carleton University, Ottawa, Ontario, Canada. We used laboratory-reared *Gryllus* offspring (*G. texensis*: first and second generation; *G. assimilis*: first generation; *G. veletis*: first generation) for this experiment. We do not know the number of generations for which *A. domesticus* had been in culture prior to the start of our experiment. Crickets were housed in large species-specific colonies preceding the start of the experiment. These communal 68 litres plastic containers each had a 10 cm × 15 cm wire-mesh covered hole cut in the lid to provide light and air. Temperature was controlled at 28 ± 2°C (mean ± SE; range = 24–32°C within each day) and crickets were kept on a 12:12 h light:dark cycle with lights on at 0700 h. All four species were provided with ad libitum powdered food (Harlan Teklad Rodent diet # 8604, Indianapolis, Indiana, U.S.A.) and water. Food and water were checked daily and replaced as required. All crickets were also provided with egg cartons for shelter. Colonies containing sexually mature crickets were provided with moist soil for egg laying.

At the start of the experiment, we checked the colonies daily to ensure all newly molted adult males were removed. Each newly molted adult male was placed individually into a 500 mL plastic container (11 cm diameter × 7 cm height) containing 1 fluid ounce (30 mL) plastic cup filled with gravel and water, a crumpled piece of paper towel for shelter and 1″× 1″ (2.54 × 2.54 cm) plastic food dish. Crickets were examined every 2 days to ensure they were still alive and to replenish their food and water. Males were weighed at 1-week post-eclosion using a Denver Instruments balance (Pinnacle Series model PI-314; precision = ± 0.1 mg; Denver Instruments, Bohemia, New York, U.S.A.).

### Quantifying mate signaling

**Figure 1:**
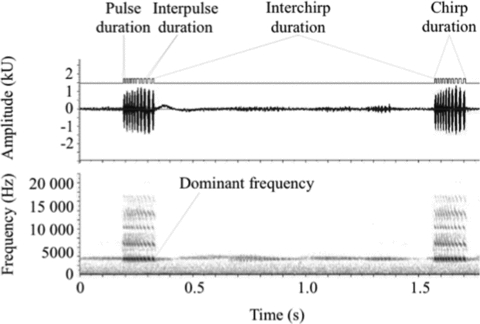
Signaling traits of the long-distance mate attraction signal of *Gryllus assimilis*.

Males were placed, still in their individual containers, into an electronic acoustic recording system (EARs II). A microphone allowed each cricket to have all of its acoustic mate attraction signals monitored throughout its time in the EARs II. Each cricket's microphone was continuously monitored in real time using a computer program (CricketSong software developed for the Bertram laboratory by Cambridge Electronic Design Ltd., Unit 4, Science Park, Milton Road, Cambridge, UK). CricketSong analyzed the separate signaling components by calculating the hourly averages for the following fine-scale temporal and spectral properties: pulse duration (PD) (ms), interpulse duration (IPD) (ms), pulses per chirp, chirp duration (CD) (ms), pulse rate (number of pulses/s), interchirp duration (ICD) (ms), chirp rate (#chirps/min), carrier frequency (CF) (Hz), and amplitude (AMP) (dB; Fig. 1; Table 1). CricketSong also determined each male's total time spent signaling each hour (min), which reflects each male's hourly signaling effort. Each male's acoustic signaling traits were quantified for 24 h a day, over the entire time that the male spent in the EARs II (8–14 days post adult-molt = 7 consecutive days).

**Table 1:** Descriptive statistics for all acoustic signal components. Trait abbreviations listed to the right of the trait name

Trait	Species	*N*	Mean	SE	CV	*r*	*F*	*P*	R^2^_adj_	Diff.
Signaling time (min) TSC	*A. domesticus*	117	22.14	1.39	67.74	0.6499	66.76	<0.0001	0.41	A
	*G. assimilis*	85	181.01	16.86	85.90	0.9655				B
	*G. texensis*	35	16.44	4.59	165.38	0.4329				A
	*G. veletis*	51	170.93	18.60	77.70	0.9074				B
Pulse duration (ms) PD	*A. domesticus*	100	14.87	0.46	31.08	0.9836	71.95	<0.0001	0.45	A
	*G. assimilis*	82	9.69	0.16	14.78	0.9574				B
	*G. texensis*	25	7.73	0.27	17.29	0.7381				C
	*G. veletis*	49	15.07	0.23	10.83	0.9452				A
Interpulse duration (ms) IPD	*A. domesticus*	100	63.79	0.63	9.91	0.9408	2197.07	<0.0001	0.96	A
	*G. assimilis*	82	16.19	0.20	11.46	0.9345				B
	*G. texensis*	25	14.53	0.28	9.77	0.8828				B
	*G. veletis*	49	31.62	0.39	8.60	0.8595				C
Chirp duration (ms) CD	*A. domesticus*	100	82.47	0.84	10.22	0.9184	140	<0.0001	0.62	A
	*G. assimilis*	82	114.63	2.16	17.04	0.9431				B
	*G. texensis*	25	450.30	51.99	57.73	0.8604				C
	*G. veletis*	49	121.36	3.11	17.94	0.9660				B
Interchirp duration (ms) ICD	*A. domesticus*	100	1104.04	60.66	54.94	0.9385	72.57	<0.0001	0.46	A
	*G. assimilis*	82	1571.87	52.36	30.16	0.8789				B
	*G. texensis*	25	354.94	27.33	38.50	0.9889				C
	*G. veletis*	49	511.42	24.80	33.94	0.8790				C
Carrier frequency (Hz) CF	*A. domesticus*	100	4502.00	29.17	6.48	0.9330	358.12	<0.0001	0.81	A
	*G. assimilis*	82	3780.25	33.74	8.08	0.9503				B
	*G. texensis*	25	5238.55	44.32	4.23	0.6119				C
	*G. veletis*	49	5309.56	38.75	5.11	0.9556				C
Amplitude (dB) AMP	*A. domesticus*	100	50.54	1.16	22.98	0.9597	67.13	<0.0001	0.44	A
	*G. assimilis*	82	47.22	1.35	25.85	0.8044				A
	*G. texensis*	25	58.16	1.85	15.91	0.6072				B
	*G. veletis*	49	73.91	1.21	11.45	0.8526				C
Pronotum area (mm^2^)	*A. domesticus*	117	8.64	0.14	16.44		401.76	<0.0001	0.81	A
	*G. assimilis*	85	19.62	0.32	14.91					B
	*G. texensis*	35	18.66	0.56	17.76					B
	*G. veletis*	51	15.47	0.27	12.34					C
Wet mass (mg)	*A. domesticus*	117	253.74	4.38	17.85		150.26	<0.0001	0.63	A
	*G. assimilis*	85	482.19	11.02	20.82					B
	*G. texensis*	35	393.73	17.35	24.93					C
	*G. veletis*	51	431.64	10.56	16.41					C

CV = coefficient of variation; *r* = repeatability; Diff. = species with different letters are statistically different using post hoc Tukey's test; R^2^_adj_ = adjusted R^2^; SE = Standard Error; F and P = outputs from an analysis of variance.

During acoustic recording, pulse onset was determined when the AMP recorded by the microphone passed a species-specific threshold (e.g., 40.0 dB for *G. assimilis*). This threshold was also adjusted dynamically by CricketSong to account for individuals that signal at higher than average AMPs. For these very loud individuals, the threshold was raised to a level proportional to the AMP of the pulse and then back to the original value within 1–8000 ms (the exact rate of decay was proportional to the size of the pulse, allowing the system to be self-scaling). For very quiet individuals, the minimum threshold was manually reset to values below the species-specific value. For more details of the recording settings and thresholds, see Fitzsimmons and Bertram (2011).

Each male was acoustically isolated from the others while in the EARs II. Males were placed in their individual containers into Styrofoam™ enclosures with 7-cm thick walls lined with 3.5-cm thick acoustic foam. Inside each cricket's Styrofoam enclosure (but outside the cricket's plastic container), a single light-emitting diode provided each male with the same 12:12 h light:dark cycle under which they were raised.

To calculate daily averages for signaling traits, hourly averages were weighted by the number of pulses produced in the hour and then averaged over each 24 h period. In this way, hours in which males signaled a lot contributed more heavily to the daily average than hours in which males signaled for only a few seconds. We included this weighting protocol so that each pulse of sound contributed equally to the daily averages. Total time spent signaling each day was calculated by summing the number of minutes spent signaling each hour. Time spent signaling was averaged across days without any form of weighting to calculate average daily time spent signaling.

To reduce the potential for multicollinearity, we did not include pulses per chirp and pulse rate in P because these traits represent linear combinations of other measured traits. Instead, we utilized only seven signaling traits to calculate the P matrices (time spent signaling [TSC], PD, IPD, CD, ICD, CF, and AMP; Fig. 1).

### Mate signaling repeatability

We used a Box-Cox transformation in JMP v. 8.0.2 (SAS Institute, Cary, NC) to normalize time spent signaling. All other signaling traits were normally distributed. We assessed within-individual variation in mating signals by quantifying the repeatability of all signaling traits using the intraclass correlation coefficients following Falconer and Mackay (1996).

### Estimating phenotypic variance–covariance matrices

We estimated individual-level phenotypic variance– covariance matrices via restricted maximum likelihood (REML) as implemented in the MIXED procedure of SAS v. 9.2 (SAS Institute, Cary, NC). We employed a repeated measures approach as described in McGuigan and Blows (2007). A single, global P matrix (i.e., across all four species) was first estimated using the following multivariate mixed model:


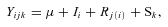
(1)

where *Y* is the observed signaling trait of replicate measurement *R* nested within individual *I*. Fixed effects included the intercept (µ) and species (S). Because species were coded as a fixed effect, our inferences should be restricted to the four species included in our study; discussion about other cricket species is speculative.

Before analysis, the seven signaling traits were individually standardized (∼*N*(0,1)) globally (i.e., across species). Due to convergence problems when attempting to estimate an unconstrained covariance matrix at the individual level (P), the model was fit via a factor-analytic approach (Kirkpatrick and Meyer 2004; Hine and Blows 2006), employing three dimensions at the individual level, which is the maximum number for which convergence could be obtained, and an unconstrained variance–covariance matrix at the replicate level.

As a direct test of Hypothesis 1, that P differ among species, we used a likelihood-ratio test to compare the fit of the above model (Equation 1) to one in which separate P matrices were estimated for each species by employing the “group” statement at the individual level in the SAS MIXED procedure. P matrices were estimated for each species using Equation (1), excluding the fixed effect of species (S), fit separately by species. In these analyses, signaling traits were standardized (∼*N*(0,1)) separately for each species.

Our repeated measures approach to estimating P required replicate measures of each trait from every individual. To record daily mate signaling traits, replicate measures were taken across a 1 week period and hence at different ages (7–14 days postimaginal molt). Some or all of these signaling traits may change within an individual as they age (e.g., Bertram 2000; Judge et al. 2008; Fitzsimmons and Bertram 2011; Judge 2011). Age-associated effects would contribute to variance at the replicate (*R*), and not individual (*I*) levels in Equation (1). In addition, there was little evidence of any directional change in any of the signaling traits across this 1 week span (S. Bertram, unpubl. results). Estimates of P also changed little if, in place of an unconstrained variance–covariance matrix at the replicate level, we instead employed an autoregressive covariance structure in which the correlation between two measurements decreased exponentially as the length of time between them increased (H. Rundle, unpubl. results).

### Quantifying dimensionality

To test Hypothesis 2, those P matrices are of full dimensionality, we used factor-analytic modeling to determine the dimensionality of P within each species. We constrained the individual-level variance–covariance matrix to be from zero through seven dimensions, employing an unconstrained variance–covariance matrix at the replicate level in all cases. A series of nested likelihood-ratio tests were used to determine whether excluding each dimension significantly worsened the fit of the model (Hine and Blows 2006; McGuigan and Blows 2007).

### Comparing phenotypic variance–covariance matrices among species

Differences among species in phenotypic covariance structure were characterized via a comparison of the eigenstructure of P. We employed a formal method of subspace comparison of P matrices originally developed by Krzanowski (1979), which has been outlined in detail elsewhere (Blows et al. 2004; McGuigan and Blows 2007). In brief, the Krzanowski method is a geometric approach that provides a bounded measure of the overall similarity in orientation of two subspaces and is not restricted to any type of original matrix. For a given pair of species, we created matrices A and B, where the columns of A are the first three eigenvectors of P for the first species and the columns of B are the first three eigenvectors of P for the second species. Note that the number of columns in each matrix A and B (here equal to three) cannot exceed half the number of columns of P or the method will recover common dimensions between the subspaces (Blows et al. 2004). The matrix S is calculated as:


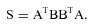
(2)

The similarity of the two subspaces defined by matrices A and B is then assessed as the sum of the eigenvalues of matrix S, the upper bound of which here equals three. Dividing the sum of eigenvalues of S by three, therefore, yields a similarity coefficient ranging between zero, indicating that the three eigenvectors of each subspace are orthogonal, to one, indicating a set of eigenvectors with identical orientation. This subspace comparison was performed between all six unique combinations (6 = (^4^_2_)) of the four species and the resulting values were assembled into a symmetrical 4 × 4 similarity matrix § characterizing the among-species variation in P. The eigenstructure of § was then examined via diagonalization to determine the dominant axes of the among-species variation in P.

Patterns in the among-species variation in P were explored by calculating the correlation between the similarity matrix § and a matrix of phylogenetic distances between these four species, as outlined in Rundle et al. (2008), with significance determined using Mantel tests employing 10,000 randomizations (Mantel 1967). Phylogenetic distances were calculated from a slightly modified tree originally constructed by Huang et al. (2000) using two mitochondrial DNA loci. Because branch length data were unavailable from Huang et al. (2000) or directly from the authors, Bertram et al. (2011a) measured the branch lengths of Huang et al.'s Figure 3 and then replicated the tree using Mesquite version 2.74 (Maddison and Maddison 2010). This correlation analysis informed us whether differences in P were associated with phylogenetic distance.

To compare the dominant axis of among-individual phenotypic variance (i.e., *p*_max_, the eigenvector of P associated with the largest eigenvalue) among species, we calculated the vector correlation of *p*_max_ between each of the six unique pairwise combinations of the four species. Vector correlations were calculated as the dot product of *p*_max_ from a pair of species, each standardized to unit length (Rundle et al. 2008; Blows and Walsh 2009). These correlations represent a measure of the overall similarity in the multivariate direction of *p*_max_ between two species and range from –1 (indicating the same multivariate trait combinations, but oriented 180˚ from one another) to +1 (indicating the same trait combination and direction), with zero indicating orthogonal eigenvectors.

### Is signaling correlated with size or condition?

To test Hypothesis 3, that variation in signaling traits reflects underlying variation in body size, mass, and/or condition, we first scored individuals, separately by species, for the first eigenvector of the P matrix (*p*_max_). Best linear unbiased predictors (BLUPs) were then estimated at the individual level for this trait in a univariate version of Equation (1), again excluding the fixed effect of species (S) and fit separately by species. An index of body size was obtained by quantifying the area of each cricket's pronotum at the end of the experiment. Pronotum measurements were made using AxioVisionLE v4.8 from highly magnified photographs taken with a Zeiss Axio Observer inverted dissecting microscope (Carl Zeiss; magnification: ∼8.5×, resolution: ∼1.60µm). Body mass was measured at day 7 (prior to the recording of the signaling traits). As a measure of condition, we used the residuals of a regression of body mass on pronotum area, again fit separately by species. Because repeated measurements of pronotum area and body mass were not available, for each species the individual BLUPs for *p*_max_ were separately regressed directly onto individual values of pronotum area and condition to determine whether among-individual variation in *p*_max_ scores was associated with any of these traits.

## Results

Across species, mate signaling was repeatable within individuals (Table 1; overall mean ± SE = 0.87 ± 0.02), with repeatability scores ranging from 0.91 to 0.93 for PD, IPD, pulses per chirp, and CD. Repeatability scores were slightly lower for CF and AMP (mean = 0.86 and 0.81, respectively), with signaling time exhibiting the lowest repeatability scores (mean = 0.74). There was extensive variation among males in their mate signaling behavior (Table 1). Coefficients of variation were greatest for signaling time (mean = 99) and were large for PD, CD, and AMP (means of 19–34). Coefficients of variation were smallest for IPD and CF (means of 6–12).

We found strong support for correlations between signaling components. Males that signaled with longer PDs produced signals (a) with longer CDs (*A. domesticus* and *G. assimilis*), (b) at lower carrier frequencies (*A. domesticus*, *G. assimilis*, and *G. veletis*), and (c) at higher AMPs (*A. domesticus*, *G. assimilis*, and *G. texensis*) (Table 2). Males that signaled at higher AMPs also signaled with longer CDs (*A. domesticus*, *G. assimilis*, and *G. texensis*). Furthermore, males that signaled more (i.e., had higher signaling times) also produced (d) louder chirps that had (e) shorter IPDs (*A. domesticus*, *G. assimilis*, and *G. veletis*), (f) longer CDs (*G. assimilis* and *G. texensis*), and (g) lower carrier frequencies (*A. domesticus* and *G. assimilis*) (Table 2). Together these findings indicate positive correlations between the quality and quantity of mating signals.

**Table 2:** Phenotypic variance–covariance matrices (P matrices) of seven acoustic signaling traits for four cricket species, as estimated from the individual-level variance–covariance matrix in a multivariate mixed model fit separately by species. Phenotypic variances are given along the diagonal (bold) and covariances (lower left) and correlations (upper right, in italics) are given on the off-diagonals. *p*_max_, *p*_2_, and *p*_3_ are the first three eigenvectors of P, respectively. Trait abbreviations are given in Table 1

	AMP	CD	CF	ICD	IPD	PD	TSCX^1^	*p*_max_	*p*_2_	*p*_3_
*A. domesticus*										
AMP	**0**.**795**	*0.271*	−*0.250*	−*0.302*	−*0.430*	*0.669*	*0.766*	−0.466	−0.234	0.369
CD	0.193	**0**.**635**	−*0.413*	*0.266*	*0.362*	*0.652*	*0.229*	−0.276	0.500	0.270
CF	−0.221	−0.326	**0**.**981**	−*0.085*	*0.148*	−*0.521*	−*0.349*	0.416	−0.300	0.786
ICD	−0.190	0.149	−0.059	**0**.**498**	*0.359*	−*0.030*	−*0.324*	0.078	0.384	−0.105
IPD	−0.338	0.255	0.130	0.224	**0**.**779**	−*0.197*	−*0.597*	0.241	0.601	0.328
PD	0.594	0.518	−0.513	−0.021	−0.173	**0**.**992**	*0.575*	−0.574	0.196	0.226
TSCX	0.497	0.133	−0.252	−0.167	−0.384	0.418	**0**.**531**	−0.375	−0.243	0.057
*G. assimilis*										
AMP	**0**.**718**	*0.662*	−*0.462*	−*0.485*	−*0.249*	*0.546*	*0.647*	0.509	0.008	0.333
CD	0.358	**0**.**407**	−*0.663*	−*0.271*	*0.094*	*0.880*	*0.565*	0.363	0.327	0.043
CF	−0.277	−0.299	**0**.**498**	*0.139*	*0.039*	−*0.746*	−*0.379*	−0.329	−0.380	0.503
ICD	−0.298	−0.125	0.071	**0**.**527**	*0.376*	−*0.265*	−*0.655*	−0.324	0.371	−0.545
IPD	−0.140	0.040	0.018	0.181	**0**.**441**	*0.157*	−*0.650*	−0.171	0.564	0.544
PD	0.307	0.373	−0.349	−0.127	0.069	**0**.**440**	*0.442*	0.348	0.411	−0.006
TSCX	0.448	0.295	−0.219	−0.388	−0.353	0.240	**0**.**667**	0.495	−0.353	−0.204
*G. texensis*										
AMP	**0**.**486**	*0.662*	−*0.162*	−*0.628*	*0.010*	*0.826*	*0.797*	0.501	0.037	−0.161
CD	0.360	**0**.**607**	*0.164*	−*0.522*	−*0.081*	*0.416*	*0.859*	0.459	0.281	0.256
CF	−0.090	0.102	**0**.**641**	−*0.253*	−*0.432*	−*0.520*	*0.070*	−0.133	0.635	0.503
ICD	−0.282	−0.262	−0.131	**0**.**415**	*0.166*	−*0.440*	−*0.830*	−0.346	−0.262	−0.063
IPD	0.006	−0.052	−0.282	0.087	**0**.**665**	*0.349*	−*0.061*	0.092	−0.585	0.787
PD	0.450	0.253	−0.326	−0.221	0.222	**0**.**611**	*0.625*	0.523	−0.289	−0.168
TSCX	0.274	0.330	0.028	−0.263	−0.024	0.241	**0**.**243**	0.345	0.148	0.068
*G. veletis*										
AMP	**0**.**734**	−*0.396*	*0.522*	−*0.697*	−*0.590*	−*0.112*	*0.621*	−0.433	−0.073	0.414
CD	−0.321	**0**.**895**	−*0.169*	*0.462*	*0.617*	*0.031*	−*0.168*	0.352	0.573	0.487
CF	0.419	−0.150	**0**.**878**	−*0.276*	−*0.406*	−*0.432*	*0.404*	−0.365	0.519	0.049
ICD	−0.524	0.384	−0.227	**0**.**772**	*0.576*	*0.247*	−*0.535*	0.423	0.185	−0.171
IPD	−0.444	0.513	−0.335	0.445	**0**.**774**	*0.393*	−*0.529*	0.455	0.102	0.274
PD	−0.084	0.026	−0.356	0.191	0.304	**0**.**774**	−*0.209*	0.230	−0.585	0.539
TSCX	0.428	−0.128	0.304	−0.378	−0.375	−0.148	**0**.**648**	−0.342	0.100	0.441

1 TSC has been normalized using a Box-Cox transformation to yield TSCX.

We found evidence to support Hypothesis 1, that species exhibit different P matrices. First, individual-level P matrices were estimated separately for each species (Table 2) because the model that permitted species specific (i.e., separate), as opposed to a shared variance–covariance structure, exhibited a significantly improved fit [χ^2^ = 3892, df = 54, *P* < 0.0001; Akaike Information Criterion (AIC), shared: 11,754.4, separate: 7970.4]. Variation between individuals accounted for 51.3, 68.2, 52.6, and 75.6% of the total variance in this suite of traits in *A. domesticus*, *G. assimilis*, *G. texensis*, and *G. veletis*, respectively, with the remaining variance occurring among replicate measurements within individuals (the latter likely including mostly true biological variation within-individuals, such as age-related changes in signaling along with some measurement error). Second, *G. texensis* differed from the other three species in that factor-analytic modeling only supported the existence of five of the seven dimensions; modeling supported the existence of all seven dimensions in the other three species (discussed in detail the next paragraph). Third, *G. veletis* differed substantially from the other three species in the first eigenvector (with the largest eigenvalue) of P. The first eigenvector of P (*p*_max_) accounted for 46–55% of the total individual-level variation within each species (Table 3). While *p*_max_ was largely shared among three of the species—*A. domesticus*, *G. assimilis*, *G. texensis*—with vector correlations ranging in absolute value from 0.85 to 0.93 (Table 4), *p*_max_ of *G. veletis* differed substantially from the other three species, with vector correlations ranging in absolute value from 0.09 to 0.28 (Table 4).

**Table 3:** Model fit statistics of the effective number of dimensions of the individual-level phenotypic variance–covariance matrices (P matrices) for each species, as determined from a series of nested likelihood-ratio tests employing a factor-analytic model. The percent of the total variance (% var) was calculated from a full-dimensionality (unconstrained) model at the individual level. Significant *P*-values in bold

No. of dimensions	% var	No. of traits	-2LL	AIC	*P*-value
*A. domesticus*					
0	—	28	9660.3	9716.3	—
1	46.9	35	8810.2	8880.2	**<0.0001**
2	24.9	41	8251.8	8333.8	**<0.0001**
3	12.7	46	7868.5	7960.5	**<0.0001**
4	6.8	50	7618.4	7716.4	**<0.0001**
5	4.0	53	7430.3	7534.3	**<0.0001**
6	3.2	55	7310.7	7420.7	**<0.0001**
7	1.4	56	7261.5	7373.5	**<0.0001**
*G. assimilis*					
0	—	28	2849.0	2905.0	—
1	55.0	35	2722.1	2792.1	**<0.0001**
2	22.7	41	2604.0	2684.0	**<0.0001**
3	8.8	46	2531.8	2621.8	**<0.0001**
4	7.0	50	2482.3	2580.3	**<0.0001**
5	4.4	53	2448.2	2554.2	**<0.0001**
6	1.4	55	2420.6	2530.6	**<0.0001**
7	0.7	56	2416.5	2528.5	**0**.**0436**
*G. texensis*					
0	—	28	1695.6	1751.6	—
1	46.1	35	1601.0	1669.0	**<0.0001**
2	31.4	41	1533.7	1613.7	**<0.0001**
3	11.2	46	1474.3	1566.3	**<0.0001**
4	6.8	50	1451.3	1551.3	**0**.**0001**
5	3.3	53	1442.5	1548.5	**0**.**0326**
6	1.0	55	1440.7	1560.4	0.3970
7	0.1	56	1440.7	1552.7	0.8557
*G. veletis*					
0	—	28	5417.4	5473.4	—
1	49.5	35	4882.2	4952.2	**<0.0001**
2	17.7	41	4441.8	4523.8	**<0.0001**
3	12.3	46	4121.7	4211.7	**<0.0001**
4	9.2	50	3876.0	3976.0	**<0.0001**
5	5.8	53	3717.9	3823.9	**<0.0001**
6	3.3	55	3599.4	3709.4	**<0.0001**
7	2.3	56	3532.6	3644.6	**<0.0001**

**Table 4:** Similarity matrix, §, comparing the individual-level phenotypic variance–covariance matrices (P matrices) among all pairwise combinations of the four species of crickets. Values below the diagonal are Krzanowski (1979) subspaces comparison of the first three eigenvectors of P and are standardized to range from zero, indicating noncoincident subspaces (i.e., no similarity), to one, indicating identical subspaces. Values above the diagonal are the vector correlation of *p*_max_, ranging from –1, indicating that the two eigenvectors are oriented 180˚ from one another, to +1, indicating two identical eigenvectors, with zero indicating orthogonal vectors

	*A. domesticus*	*G. assimilis*	*G. texensis*	*G. veletis*
*A. domesticus*		−0.926	−0.849	0.092
*G. assimilis*	0.795		0.915	−0.277
*G. texensis*	0.735	0.706		−0.109
*G. veletis*	0.753	0.613	0.711	

We also found support for Hypothesis 2, that the phenotypic variance–covariance matrices are full dimensionality. Factor-analytic modeling supported the existence of all seven dimensions in the P matrices of three of the species (*A. domesticus*, *G. assimilis*, and *G. veletis*), and five of the seven dimensions in *G. texensis* (Table 3). This indicates that, to a large extent, each measured signaling trait makes some unique contribution to the population-level phenotypic variance among individuals. That only five of the seven dimensions are supported in *G. texensis* suggests that this species may be an outlier relative to the other three.

Among the seven traits we analyzed, variance inflation factors were low, ranging from one to two with a single outlier value of five, indicating minimal multicollinearity (Chatterjee and Hadi 2006) and suggesting that we appropriately chose traits. Condition numbers (ratio of maximal to minimal eigenvalues) were also small, indicating the absence of strong multicollinearity (Chatterjee and Hadi 2006; Visanuvimol and Bertram 2010).

**Figure 2:**
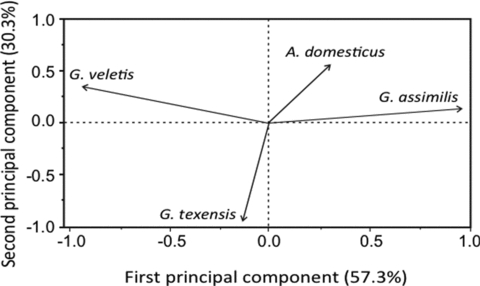
Two-dimensional bi-plot depicting the dominant axes of variation in the among-species similarity matrix, **§**, comparing subspaces of P (data derived from Table 4, below the diagonal).

A comparison of the subspaces defined by the first three eigenvectors of P among all species pairs using the method of Krzanowski (1979) yielded values ranging from 61% similarity (between *G. assimilis* and *G. veletis*) to 80% similarity (between *G. assimilis* and *A. domesticus*), indicating a moderately high degree of overall similarity in the eigenstructure of the three-dimensional subspaces of P of each species (Table 4). The subspaces defined by the first three eigenvectors of P within each species accounted for the majority of the individual-level variation, representing 85, 87, 89, and 80% of the total variance in P in *A. domesticus*, *G. assimilis*, *G. texensis*, and *G. veletis*, respectively (Table 3). The eigenstructure of §, the similarity matrix of Krzanowski subspace comparisons (Table 4), which reflects patterns in the among-species variation in P, arrayed the species roughly equidistantly from one another. *Gryllus veletis* differed the most from the other species, while *A. domesticus* and *G. assimilis* were the most similar overall (Fig. 2). There was no indication of a negative association between this similarity matrix and a matrix of phylogenetic distances among the four species, as would be expected if P differed to a greater extent between more distantly related species. Rather, the observed correlation between these matrices was positive (*r* = 0.768), although not significantly more extreme than expected by chance (Mantel test, *P* = 0.077).

There was limited evidence to support Hypothesis 3, that the eigenvector associated with the largest eigenvalue of each P matrix is correlated with size and/or condition, insofar as the first eigenvector of P, in part, reflected among-individual variation in size. BLUPs for *p*_max_ scores were significantly associated with variation in body size (pronotum area) in *A. domesticus* and *G. veletis* (Table 5; the contrasting sign of this relationship in *A. domesticus* relative to *G. veletis* reflects the opposite orientation of *p*_max_ in this species.) There is no evidence of any association between *p*_max_ BLUPs and body size in *G. assimilis* and *G. texensis*, nor is there evidence from any of the four species for an association with condition (Table 5).

**Table 5:** Model fit statistics for the linear regression of individual-level BLUPs for *p*_max_ on body size (pronotum area) and condition (measured as the residuals of the regression of body mass on pronotum area), separately by species. Significant *P*-values are bolded

	df	Slope (*P*-value)	*r*^2^
*A. domesticus*			
Size	1.95	**−0.345 (<0.001)**	0.255
Condition	1.95	−0.007 (0.136)	0.023
*G. assimilis*			
Size	1.28	0.003 (0.941)	0.000
Condition	1.27	0.002 (0.438)	0.022
*G. texensis*			
Size	1.23	0.048 (0.289)	0.049
Condition	1.21	0.003 (0.156)	0.094
*G. veletis*			
Size	1.45	**0**.**191 (0.012)**	0.132
Condition	1.40	−0.002 (0.603)	0.007

## Discussion

An important goal in evolutionary biology is to identify what constraints prevent populations from responding fully to selection (McGuigan and Blows 2007). Most researchers have tackled this problem by either quantifying the genetic correlations among traits or examining the relationships between selection and additive genetic variance (Blows and Higgie 2003). However, the distribution of populations in phenotypic space (the P matrix) is also important as it determines the combinations of traits available to selection. The dimensionality of P may place an upper bound on the dimensionality of the genetic variance–covariance matrix (G) (McGuigan and Blows 2007). Several robust studies have also revealed that P may provide additional information about the structure of G (Cheverud 1988; Roff 1995; Waitt and Levin 1998; Dochtermann 2011).

We determined that selection was not limited in the directions in which it could act upon male cricket long-distance mate signaling traits. Factor-analytic modeling supported the existence of all seven dimensions of P in three of the four species studied (*A. domesticus*, *G. assimilis*, and *G. veletis*). These findings suggest that each of the seven measured signaling traits made some unique contribution to the population-level phenotypic variance among individuals. As such, the potential exists for all seven signaling traits to experience selection, with minimal potential for evolutionary constraints. Selection may be able to shift each population toward its adaptive peak, regardless of the position of an adaptive optimum, provided that the signaling traits we measured exhibit genetic variance.

While our results suggest that all of the measured traits capture some unique information and that all combinations of traits exist in the population, our results do not imply that researchers should study these univariate signaling traits separately. Significant correlations still exist between pairs of traits, suggesting that selection may target trait combinations (i.e., correlational selection); univariate analyses would miss this phenomenon.

Factor-analytic modeling suggests the existence of only five of seven dimensions in the P matrix of *G. texensis*, indicating that *G. texensis* may be an outlier compared to *A. domesticus*, *G. assimilis*, and *G. veletis*. Less than full dimensionality suggests the potential for multicollinearity-–where the sixth and seventh dimensions can be expressed as some linear combination of the first five dimensions. Statistical support for only five of seven dimensions suggest evolutionary constraints may exist because only a subset of the seven traits might be available to selection. *Gryllus texensis* may therefore be evolutionarily constrained in ways that *A. domesticus*, *G. assimilis*, and *G. veletis* are not. Lack of full dimensionality also suggests that *G. texensis* may have fewer traits on which selection might act. In other words, some traits may covary with other traits. While we know what the seven traits are, we do not know what the sixth and seventh dimensions represent, other than linear combinations of the seven measured traits. Unless P matrices are diagonal, there is no easy interpretation of dimensions in terms of traits.

Less than full dimensionality in *G. texensis* may have resulted from insufficient statistical power because of a smaller sample size of *G. texensis*. Further, the sixth and seventh dimensions explained very little of the variance in all of the species studied (*A. domesticus* [4.6%], *G. assimilis* [2.1%], *G. texensis* [1.1%], and *G. veletis* [5.5%]). Even though the sixth and seventh dimensions were statistically significant for *A. domesticus*, *G. assimilis*, and *G. veletis*, these two dimensions do not differ much from the small amount of variance explained by the sixth and seventh dimensions for *G. texensis* that were not statistically significant.

In addition to the dimensionality of its P matrix, *G. texensis* differs from the other three species studied in two distinct ways. First, *G. texensis* is the only study species to be regularly attacked by the parasitoid *O. ochracea* (Diptera, Tachinidae) (Cade 1975). *Ormia ochracea* females have a large tympanic membrane on their thorax that allows them to listen for signaling male *G. texensis* (Robert et al. 1996). *Ormia ochracea* females acoustically orient to signaling male *G. texensis*, laying their larvae on and around the cricket. Fly larvae burrow into the cricket, feed on the cricket's tissue, and cause host cricket death within a week (Cade 1975; Berrill et al. 1993; Adamo et al. 1995). This parasitoid fly is thought to select against mate attraction signaling in *G. texensis* (Cade 1975; Wineriter and Walker 1990; Robert et al. 1992; Adamo et al. 1995). Second, *G. texensis* is the only species studied that produces an extremely long chirp, concatenating an average of 37 pulses into a single chirp (termed a trill in *G. texensis*). The other three species concatenate only a small number of pulses into each chirp (*A. domesticus*: 2–3 pulses; *G. assimilis*: 6–9 pulses; and *G. veletis*: 3–5 pulses). These dissimilarities provide additional reasons why *G. texensis* may differ from *A. domesticus*, *G. assimilis*, and *G. veletis* in the dimensionality of its P matrix.

Mate choice often exerts directional sexual selection on mate attraction signals (Andersson 1994). However, under some circumstances, intermediate phenotypes are most preferred (Andersson 1994; Mead and Arnold 2004; Brooks et al. 2005). Historically, female crickets have been shown to exert both stabilizing and directional selection on male acoustic mate attraction signals (Cade 1979b, 1981a; Hedrick 1986; Doherty and Callos 1991; Wagner 1996; Holzer et al. 2003; Hunt et al. 2004; Brooks et al. 2005; Bentsen et al. 2006; Bailey 2008; Rodríguez–Muñoz et al. 2010). Together, these studies provide clues for the potential effect female preference could have on the evolution of cricket mate signaling. They suggest the general trend that females exert directional selection for more conspicuous displays (louder signals, shorter interchirp intervals, longer bout durations, and more time spent signaling; Cade 1979a; Hedrick 1986; Wagner 1996; Wagner and Hoback 1999; Hunt et al. 2004; Brooks et al. 2005) and stabilizing selection for species identification traits (CF and pulse rate; Doherty and Callos 1991; Bentsen et al. 2006). When coupled with our P matrices and dimensionality analyses, these findings suggest that selection for one temporal component, such as enhanced PD, has the potential to result in responses in several other signaling traits including elevated AMP and higher nightly signaling times. That said, we recognize that a correlated response to selection arises from a genetic, not phenotypic, covariance, so a test of this hypothesis requires G matrix estimates.

For a trait to respond to selection, it requires (1) sufficient phenotypic variation for selection to act, and (2) sufficient genetic variation for selection to result in an evolutionary response. Our study reveals support for the first requirement—extensive phenotypic variation exists among individuals, especially in signaling time, AMP, and PD, suggesting the ability for these traits to experience selection. Two lines of evidence also suggest support for the second requirement-–sufficient genetic variation for an evolutionary response. First, the signaling traits examined in our study exhibited high repeatability, and repeatability sets the upper bounds of heritability (Falconer and Mackay 1996). Second, most cricket signaling traits studied to date exhibit high heritability. For example, *G. integer* has a heritability of *h*^2^ = 0.74 for calling-bout length (Hedrick 1988). Realized heritability for *G. texensis*’ signaling time was measured at *h*^2^ = 0.50–0.53 (Cade 1981b). However, Bertram et al. (2007) showed that signaling time did not exhibit significant heritability in a nearby *G. texensis* population (*h*^2^ = 0.08 ± 0.11). *Gryllus firmus*’ pulse rate displays a heritability of *h*^2^ = 0.35 (Webb and Roff 1992). Further, *Teleogryllus oceanicus*’ song duration and the proportion of the long-chirp element show a genetic basis (Simmons 2004). Future research should quantify whether signaling traits exhibit extensive genetic variation in the species and populations studied. If signaling traits are heritable, their response to selection would depend on the potential for traits to evolve independently and on the patterns of female preferences.

Dimensionality of the P matrix is important because it may set the upper limit to the dimensionality of the genetic variance–covariance matrix, G, and because it may provide additional information about the structure of G (Cheverud 1988; Roff 1995; Waitt and Levin 1998; Dochtermann 2011). Consequently, the dimensionality of P can be a useful measure for investigating the potential for genetic constraints. However, our finding of P of full (or almost full) dimensionality does not imply that G will be of full dimensionality in crickets, but only that it can be. McGuigan and Blows (2007) revealed that even though *Drosophila* wing P matrices were of full dimensionality for males and females, the corresponding G matrices were not. McGuigan and Blows (2007) quantified the dimensionality of P and G for the same 10 wing size and shape traits. While there was statistical support for all 10 phenotypic dimensions in both sexes, there was statistical support for only two genetic dimensions in males and five genetic dimensions in females. The dimensionality mismatch between P and G suggests that even though selection might result in wing size and shape shifts within generations, selection may not result in across-generational shifts, because evolution may be restricted to fewer dimensions due to the genetic covariance structure of the traits. Our future work will examine dimensionality of G in field cricket species to ascertain if crickets, like *Drosophila*, exhibit G matrices with much lower dimensionality than do the corresponding P matrices.

### Species differences

The four cricket species all exhibited statistically different P matrices from each other, supporting Hypothesis 1. *Gryllus texensis* differed from the other three species in that it lacked a P matrix of full dimensionality. Having a dimensionality of only five (out of a maximum of seven) suggests that *G. texensis* may be subject to more evolutionary constraints than *G. veletis, G. assimilis*, and *A. domesticus*, the latter three of which have P matrices of full dimensionality. In retrospect, this result is not surprising insofar as *G. texensis* experiences greater tension between natural and sexual selection for acoustic signaling traits because it is the only one of these species in which a parasitoid acoustically homes in on signaling male crickets using the same signals that attract female conspecific crickets. The phenotypic differences between *G. texensis* and the other species can also be seen in the Krzanowski subspaces comparison of the first three eigenvectors of P, not just in the six and seventh (the last two) eigenvectors.

Krzanowski subspace comparison indicated that all four cricket species had different P matrices, with *G. texensis* and *G. veletis* being the most different from others and *G. assimilis* and *A. domesticus* being most similar to one another. This result also supports the hypothesis that species exhibit different P matrices. This result falsifies the implicit null hypothesis that P primarily reflects phylogeny, in which all three *Gryllus* species would have grouped together and would have been different from *Acheta*.

The result that *G. texensis* and *G. veletis* were most different and *G. assimilis* and *A. domesticus* were most similar also falsifies the hypothesis that differences in P reflect the number of generations species were reared in the laboratory. As a domesticated cricket, *A. domesticus* has been reared in captivity for numerous generations, while *Gryllus* species were reared in captivity for only 1–2 generations. If time spent in captivity were the driving force behind the differences in P, *Gryllus* species would have grouped together and been different from *Acheta*. P matrices of field-captured crickets may, however, differ from laboratory-reared crickets because field-captured crickets will presumably vary more in their overall condition, a hypothesis requiring future testing.

*Gryllus veletis* also differed from the other three species in the makeup of its *p*_max_*. Acheta domesticus*, *G. assimilis*, and *G. texensis* exhibited high vector correlations (|p-max|>0.85), signifying strong similarities among these three species in their *p*_max_. Conversely, *G. veletis* exhibited much lower vector correlations with the other three species (|p-max|<0.28), signifying that *G. veletis* had a different *p*_max_.

### Signaling component correlations

We proposed that there would be correlations between signaling quality and quantity. Males that signaled with longer PDs, shorter IPDs, longer CDs, and lower carrier frequencies also produced the loudest signals and signaled most often throughout the day and night. These correlations suggest that the most attractive signalers also signal with the highest effort. Our findings are consistent with signaling being an honest indicator of a cricket's overall condition, given that males with the most attractive chirps also signal with the highest effort. While specific female preference functions have yet to be built for most of the species included in our study, female field crickets tend to be most attracted to chirps that have long pulses, short interpulses, are long in duration, and have shorter interchirp intervals (Hedrick 1986, 1988; Simmons 1986; Wagner et al. 1995; Gray and Cade 1999; Holzer et al. 2003; Hunt et al. 2004; Simmons 2004). The most attractive chirps are also louder, and produced at a lower CF (Alexander 1961, 1962; Cade 1980).

Our correlational findings suggest that females do not need to assess males for extended periods of time to select a mate that signals with high effort. Because signaling effort is strongly correlated with signaling quality, a female could briefly assess males to ascertain which ones signal loudest, with long PDs, more pulses per chirp, and/or longer CDs. By selecting one of these males, the female would likely be mating with a male that signals most often. This approach would minimize the female's time spent in the mating chorus, thereby reducing her energetic costs, predation risks, and lost foraging time.

### Condition and size effects

Signaling can impose dramatic costs, both in terms of energetic demands and predation risks (Prestwich and Walker 1981; Prestwich 1994; Hoback and Wagner 1997; Basolo and Alcaraz 2003). Males that maximize the conspicuousness of their mating signals in the face of these costs may exhibit higher genetic quality than less showy males (Andersson 1994). Given that sexual signals are costly to produce and maintain, the genic capture hypothesis suggests that sexual signals should exhibit condition-dependent expression (Rowe and Houle 1996; Kotiaho et al. 2001; Tomkins et al. 2004). Males in good condition may therefore simultaneously maximize several components of their signals’ conspicuousness. Because the genic capture model assumes sexually selected traits will be costly to produce, it suggests that males in poor condition should not be able to maximize the conspicuousness of their signals. Conversely, males in good condition should produce conspicuous mating signals regardless of costs. Thus, the system should foreclose cheaters. Our findings partially support these ideas; we found a strong relationship between signaling quality and signaling effort, as discussed above. Furthermore, signaling traits were dependent on body size in two of the four species studied (*A. domesticus* and *G. veletis*). We did not, however, find support for the idea that signaling traits are strongly dependent on residual mass, our measure of condition. The general trend that variation in most signaling traits was not dependent on variation in residual mass suggests that residual mass may not be an ideal indicator of condition in cricket species. Lack of a relationship between residual mass and signaling traits should not, however, be interpreted to mean that signaling traits are not influenced by condition because several studies have shown that when diet is manipulated, several signaling traits respond, either increasing with enhanced food quality or quantity, or decreasing with reduced food quality or quantity (Scheuber et al. 2003a, b; Hedrick 2005; Whattam and Bertram 2011). Future studies should explore whether dietary limitations can change the relationships between signaling traits, thereby fundamentally altering the P or G matrices.

### Concluding remarks

We employed a recently developed rigorous repeated measures approach to characterizing P matrices in order to ascertain the differences between species, number of independent traits within species, and maximal dimensionality of genetic variance–covariance matrices. *Gryllus texensis* truly are different from their congeners and even a heterogeneric gryllid, which we surmise could be due to the acoustic signals they produce attracting both mates and parasitoids. Instead of independently analyzing univariate phenotypic traits, we recommend the analysis of vectors of phenotypic traits and their variance–covariance matrices in order to better understand the evolution of these traits and taxa. Characterizing P matrices provides insight into whether phenotypic covariances may constrain selection by making certain combinations of traits unavailable. The P matrix also provides understanding about whether the measured traits capture a dimension of phenotypic variability that is unique from the other measured traits. Finally, P is of interest with respect to evolutionary constraints as it places an upper limit on the dimensionality of the G matrix, thereby affecting the response to selection. How P differs among species has received little attention, but should be considered of interest, both in terms of how it arises and how it may subsequently affect their evolution and divergence.
